# 2-Methyl­sulfanyl-1,2,4-triazolo[1,5-*a*]quinazoline-5(4*H*)-thione

**DOI:** 10.1107/S1600536813004881

**Published:** 2013-02-23

**Authors:** Rashad Al-Salahi, Mohamed Marzouk, Mohamed A. Al-Omar, Abd El-Galil E. Amr, Seik Weng Ng, Edward R. T. Tiekink

**Affiliations:** a Department of Pharmaceutical Chemistry, College of Pharmacy, King Saud University, Riyadh 11451, Saudi Arabia; b Drug Exploration & Development Chair (DEDC), College of Pharmacy, King Saud University, Riyadh 11451, Saudi Arabia; c Applied Organic Chemistry Department, National Research Center, Dokki 12622, Cairo, Egypt; d Department of Chemistry, University of Malaya, 50603 Kuala Lumpur, Malaysia; e Chemistry Department, Faculty of Science, King Abdulaziz University, PO Box 80203 Jeddah, Saudi Arabia

## Abstract

In the title compound, C_10_H_8_N_4_S_2_, comprising fused six-, six- and five-membered rings, the mol­ecule is close to being planar (r.m.s. deviation of the non-H atoms = 0.041 Å). The S-bound methyl group is folded away from the single N atom of the triazole ring and the NH group of the six-membered ring, allowing for the formation of centrosymmetric eight-membered {⋯HNCN}_2_ synthons in the crystal. The resulting inversion dimers are connected into supra­molecular stacks aligned along the *b*-axis direction by π–π inter­actions [centroid–centroid distances = 3.6531 (12) and 3.7182 (12) Å].

## Related literature

For background to the biological activity of triazoloquinazolines, see: Pierce *et al.* (2004 ▶); Al-Salahi & Geffken (2010 ▶, 2011 ▶); Al-Salahi *et al.* (2011 ▶, 2013 ▶).
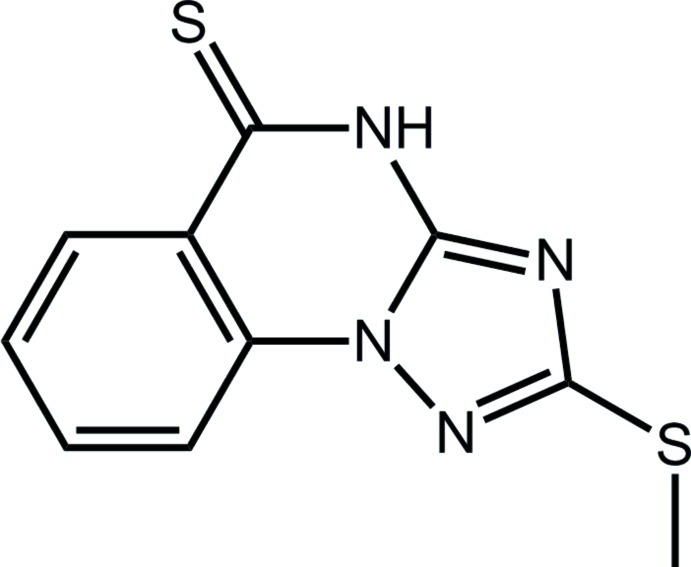


## Experimental

### 

#### Crystal data

C_10_H_8_N_4_S_2_
*M* *_r_* = 248.32
Monoclinic, 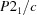

*a* = 10.5414 (11) Å
*b* = 4.9335 (6) Å
*c* = 20.0943 (19) Å
β = 99.127 (10)°
*V* = 1031.79 (19) Å^3^
*Z* = 4
Mo *K*α radiation
μ = 0.49 mm^−1^
*T* = 295 K
0.30 × 0.15 × 0.05 mm

#### Data collection

Agilent SuperNova Dual diffractometer with an Atlas detector
Absorption correction: multi-scan (*CrysAlis PRO*; Agilent, 2011 ▶) *T*_min_ = 0.864, *T*_max_ = 1.000
5096 measured reflections
2389 independent reflections
1667 reflections with *I* > 2σ(*I*)
*R*_int_ = 0.035

#### Refinement

*R*[*F* ^2^ > 2σ(*F* ^2^)] = 0.040
*wR*(*F* ^2^) = 0.098
*S* = 0.93
2389 reflections
149 parameters
1 restraint
H atoms treated by a mixture of independent and constrained refinement
Δρ_max_ = 0.28 e Å^−3^
Δρ_min_ = −0.29 e Å^−3^

### 

Data collection: *CrysAlis PRO* (Agilent, 2011 ▶); cell refinement: *CrysAlis PRO*; data reduction: *CrysAlis PRO*; program(s) used to solve structure: *SHELXS97* (Sheldrick, 2008 ▶); program(s) used to refine structure: *SHELXL97* (Sheldrick, 2008 ▶); molecular graphics: *ORTEP-3 for Windows* (Farrugia, 2012 ▶) and *DIAMOND* (Brandenburg, 2006 ▶); software used to prepare material for publication: *publCIF* (Westrip, 2010 ▶).

## Supplementary Material

Crystal structure: contains datablock(s) global, I. DOI: 10.1107/S1600536813004881/hb7045sup1.cif

Structure factors: contains datablock(s) I. DOI: 10.1107/S1600536813004881/hb7045Isup2.hkl

Supplementary material file. DOI: 10.1107/S1600536813004881/hb7045Isup3.cml

Additional supplementary materials:  crystallographic information; 3D view; checkCIF report

## Figures and Tables

**Table 1:** Hydrogen-bond geometry (Å, °)

*D*—H⋯*A*	*D*—H	H⋯*A*	*D*⋯*A*	*D*—H⋯*A*
N1—H1⋯N4^i^	0.87 (1)	2.07 (1)	2.931 (2)	171 (2)

Symmetry code: (i) 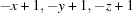.

**Fig. 1.**
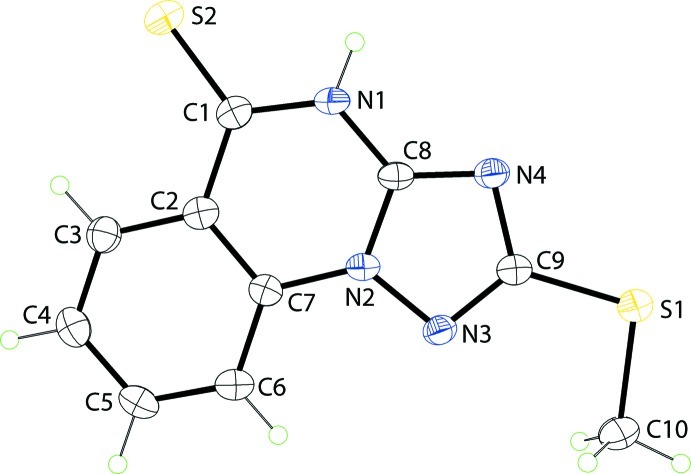
The molecular structure of (I) showing displacement ellipsoids at the 35% probability level.

**Fig. 2.**
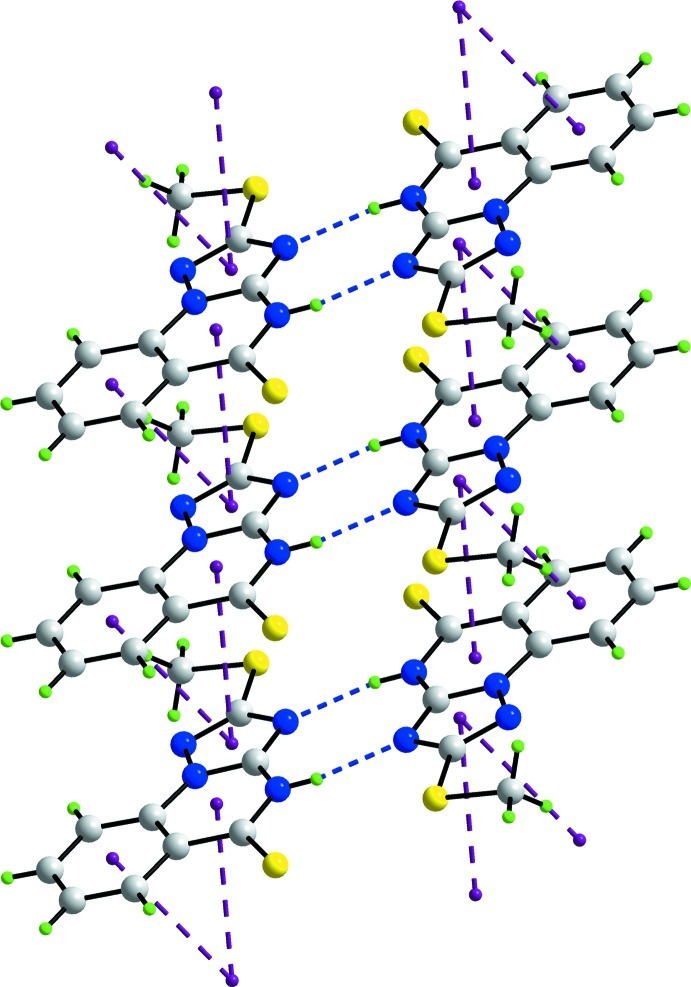
A view of the supramolecular chain along the b axis in (I) sustained by N—H···N and π—π interactions, shown as blue and purple dashed lines, respectively.

**Fig. 3.**
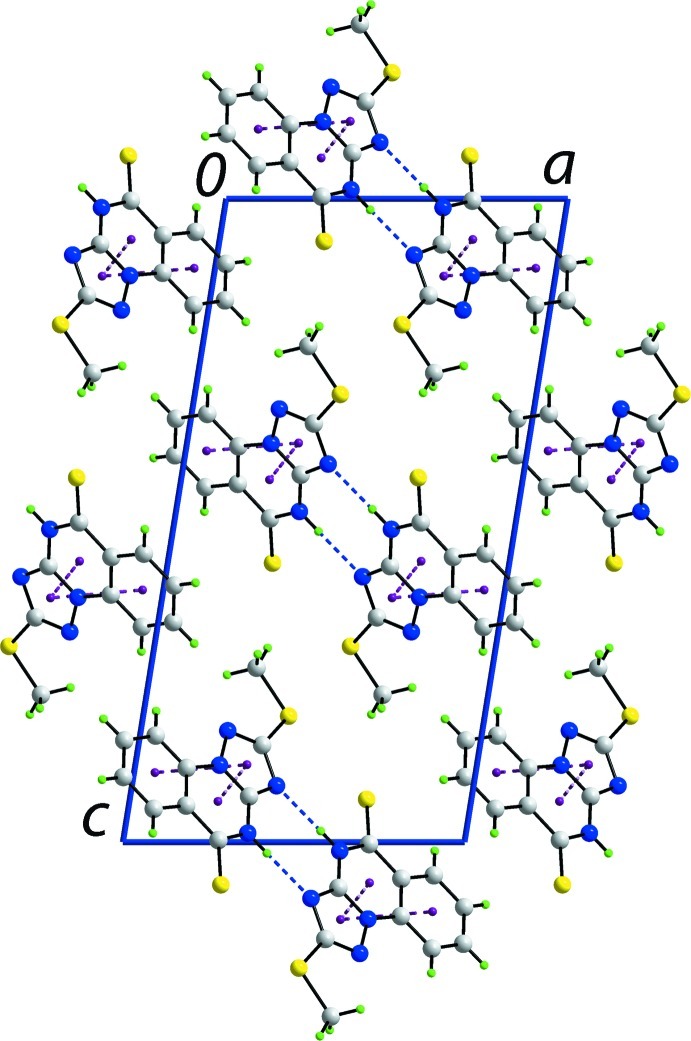
view in projection down the b axis of the crystal packing in (I). The N—H···N and π—π interactions are shown as blue and purple dashed lines, respectively.

## supplementary crystallographic information

### Comment

A series of triazoloquinazolines, which originated from *N*-cyanoimidocarbonates as synthons, has been shown to exhibit diverse biological activities. For example, 2-aminoalkyl(aryl)-[1,2,4]triazolo[1,5-*a*]quinazolin-5-ones were found to be potent proteinkinase inhibitors (Pierce *et al.*, 2004), the 2,5-dialkoxy-[1,2,4]triazolo[1,5-*a*]quinazolines have shown activity as adenosine antagonists (Al-Salahi & Geffken, 2010; Al-Salahi & Geffken, 2011; Al-Salahi *et al.*, 2011) whereas the related alkylated 1,2,4-triazolo[1,5-*a*]quinazolin-5-ones have been proven to be cytotoxic and to possess anti-inflammatory activity (Al-Salahi *et al.*, 2013). In view of the aforementioned biological activities of diverse triazoloquinazolines and in continuation of our ongoing studies dealing with the chemistry of *N*-cyanoimidocarbonates and their precursors, we report herein the results of our study of thionation of 2-methylsulfanyl-4*H*-[1,2,4]triazolo[1,5-*a*]quinazolin-5-one to obtain (I). Herein, the crystal and molecular structure of the title compound is described.

The molecular structure of (I), Fig. 1, comprises fused six-, six- and five membered rings that are co-planar with the r.m.s. deviation of the 13 non-hydrogen atoms being 0.028 Å. Indeed, the entire molecule is close to planar (r.m.s. = 0.041 Å) with the maximum deviations from the least-squares plane being 0.047 (2) Å for atom N4 and -0.072 (2) for methyl-C10. The *S*-bound methyl group is orientated towards the N3 atom and may be regarded as *anti* to the thione-S2 atom.

The most prominent feature of the crystal packing is the formation of centrosymmetric eight-membered {···HNCN}_2_ synthons, Table 1. These are connected into stacks along the *b* axis by π—π interactions whereby the triazole ring straddles the benzene [inter-centroid distance = 3.6531 (12) Å, angle of inclination = 3.04 (11)°] and pyrimidine [3.7182 (12) Å, 1.90 (10)°] rings of translationally related molecules, Fig. 2 (symmetry operation *x*, -1 + *y*, *z*]. There are no specific intermolecular interactions between stacks, Fig. 3.

### Experimental

2-Methylsulfanyl-4*H*-[1,2,4]triazolo[1,5-*a*]quinazolin-5-one (1 mmol) was refluxed with phosphorous pentasulfide (1 mmol) in absolute pyridine (5 ml) for 2 h. After cooling the reaction mixture, it was poured into ice/water. The yellow precipitate that separated was filtered off and washed thoroughly with water. Recrystallization as yellow prisms was from a mixture of toluene and DMF (8:2 *v*/*v*).

### Refinement

The C-bound H atoms were geometrically placed (C—H = 0.93–0.96 Å) and refined as riding with *U_iso_*(H) = 1.2–1.5*U_eq_*(C). The N-bound-H atom was refined with the distance retsraint N—H = 0.88±0.01 Å and free *U*_iso_.

### Crystal data

**Table:**

C_10_H_8_N_4_S_2_	*F*(000) = 512
*M**_r_* = 248.32	*D*_x_ = 1.599 Mg m^−^^3^
Monoclinic, *P*2_1_/*c*	Mo *K*α radiation, λ = 0.71073 Å
Hall symbol: -P 2ybc	Cell parameters from 1314 reflections
*a* = 10.5414 (11) Å	θ = 3.1–27.5°
*b* = 4.9335 (6) Å	µ = 0.49 mm^−^^1^
*c* = 20.0943 (19) Å	*T* = 295 K
β = 99.127 (10)°	Prism, yellow
*V* = 1031.79 (19) Å^3^	0.30 × 0.15 × 0.05 mm
*Z* = 4	

### Data collection

**Table:**

Agilent SuperNova Dual diffractometer with an Atlas detector	2389 independent reflections
Radiation source: SuperNova (Mo) X-ray Source	1667 reflections with *I* > 2σ(*I*)
Mirror monochromator	*R*_int_ = 0.035
Detector resolution: 10.4041 pixels mm^-1^	θ_max_ = 27.6°, θ_min_ = 3.1°
ω scan	*h* = −13→12
Absorption correction: multi-scan (*CrysAlis PRO*; Agilent, 2011)	*k* = −5→6
*T*_min_ = 0.864, *T*_max_ = 1.000	*l* = −22→26
5096 measured reflections	

### Refinement

**Table:**

Refinement on *F*^2^	Primary atom site location: structure-invariant direct methods
Least-squares matrix: full	Secondary atom site location: difference Fourier map
*R*[*F*^2^ > 2σ(*F*^2^)] = 0.040	Hydrogen site location: inferred from neighbouring sites
*wR*(*F*^2^) = 0.098	H atoms treated by a mixture of independent and constrained refinement
*S* = 0.93	*w* = 1/[σ^2^(*F*_o_^2^) + (0.0452*P*)^2^] where *P* = (*F*_o_^2^ + 2*F*_c_^2^)/3
2389 reflections	(Δ/σ)_max_ = 0.001
149 parameters	Δρ_max_ = 0.28 e Å^−^^3^
1 restraint	Δρ_min_ = −0.29 e Å^−^^3^

### Special details

**Table:**

Geometry. All e.s.d.'s (except the e.s.d. in the dihedral angle between two l.s. planes) are estimated using the full covariance matrix. The cell e.s.d.'s are taken into account individually in the estimation of e.s.d.'s in distances, angles and torsion angles; correlations between e.s.d.'s in cell parameters are only used when they are defined by crystal symmetry. An approximate (isotropic) treatment of cell e.s.d.'s is used for estimating e.s.d.'s involving l.s. planes.
Refinement. Refinement of *F*^2^ against ALL reflections. The weighted *R*-factor *wR* and goodness of fit *S* are based on *F*^2^, conventional *R*-factors *R* are based on *F*, with *F* set to zero for negative *F*^2^. The threshold expression of *F*^2^ > σ(*F*^2^) is used only for calculating *R*-factors(gt) *etc*. and is not relevant to the choice of reflections for refinement. *R*-factors based on *F*^2^ are statistically about twice as large as those based on *F*, and *R*- factors based on ALL data will be even larger.

### Fractional atomic coordinates and isotropic or equivalent isotropic displacement parameters (Å^2^)

**Table:**

	*x*	*y*	*z*	*U*_iso_*/*U*_eq_	
S1	0.57415 (5)	0.09061 (12)	0.69579 (3)	0.03633 (18)	
S2	0.69330 (6)	1.14441 (14)	0.43364 (3)	0.0455 (2)	
N1	0.63890 (16)	0.7597 (4)	0.51388 (8)	0.0334 (4)	
H1	0.5735 (15)	0.728 (5)	0.4828 (9)	0.052 (7)*	
N2	0.75333 (14)	0.6491 (3)	0.61961 (8)	0.0288 (4)	
N3	0.74549 (16)	0.4772 (3)	0.67308 (8)	0.0309 (4)	
N4	0.57690 (15)	0.4139 (4)	0.58757 (8)	0.0325 (4)	
C1	0.72154 (19)	0.9632 (4)	0.50311 (10)	0.0316 (5)	
C2	0.83231 (19)	1.0021 (4)	0.55680 (10)	0.0306 (5)	
C3	0.92564 (19)	1.1992 (5)	0.55164 (11)	0.0363 (5)	
H3	0.9169	1.3102	0.5138	0.044*	
C4	1.0296 (2)	1.2317 (5)	0.60125 (11)	0.0403 (6)	
H4	1.0906	1.3640	0.5970	0.048*	
C5	1.04387 (19)	1.0665 (5)	0.65804 (11)	0.0385 (5)	
H5	1.1151	1.0883	0.6914	0.046*	
C6	0.95443 (19)	0.8723 (4)	0.66552 (10)	0.0358 (5)	
H6	0.9644	0.7626	0.7036	0.043*	
C7	0.84829 (18)	0.8415 (4)	0.61517 (10)	0.0292 (5)	
C8	0.65228 (18)	0.6076 (4)	0.57102 (9)	0.0294 (5)	
C9	0.63785 (18)	0.3432 (4)	0.65066 (10)	0.0296 (5)	
C10	0.6908 (2)	0.0885 (5)	0.77103 (11)	0.0474 (6)	
H10A	0.6676	−0.0456	0.8016	0.071*	
H10B	0.6935	0.2637	0.7920	0.071*	
H10C	0.7738	0.0461	0.7599	0.071*	

### Atomic displacement parameters (Å^2^)

**Table:**

	*U*^11^	*U*^22^	*U*^33^	*U*^12^	*U*^13^	*U*^23^
S1	0.0370 (3)	0.0390 (4)	0.0320 (3)	−0.0018 (3)	0.0023 (2)	0.0034 (3)
S2	0.0496 (4)	0.0525 (4)	0.0330 (3)	−0.0007 (3)	0.0019 (3)	0.0110 (3)
N1	0.0337 (9)	0.0388 (11)	0.0247 (9)	−0.0014 (9)	−0.0046 (8)	0.0023 (8)
N2	0.0291 (8)	0.0321 (10)	0.0237 (8)	0.0019 (8)	−0.0006 (7)	0.0006 (8)
N3	0.0339 (9)	0.0321 (10)	0.0256 (9)	0.0011 (8)	0.0015 (7)	0.0028 (8)
N4	0.0328 (9)	0.0369 (11)	0.0266 (9)	0.0011 (9)	0.0007 (7)	0.0016 (8)
C1	0.0351 (11)	0.0320 (12)	0.0285 (11)	0.0054 (10)	0.0070 (9)	−0.0012 (9)
C2	0.0329 (10)	0.0314 (12)	0.0282 (11)	0.0062 (10)	0.0069 (9)	−0.0035 (9)
C3	0.0380 (11)	0.0367 (13)	0.0354 (12)	−0.0001 (11)	0.0091 (10)	−0.0006 (10)
C4	0.0366 (12)	0.0394 (14)	0.0457 (13)	−0.0061 (11)	0.0090 (10)	−0.0064 (12)
C5	0.0306 (11)	0.0436 (14)	0.0387 (12)	0.0001 (11)	−0.0024 (10)	−0.0089 (11)
C6	0.0363 (11)	0.0380 (13)	0.0313 (11)	0.0050 (10)	−0.0007 (9)	−0.0009 (10)
C7	0.0287 (10)	0.0304 (11)	0.0283 (11)	0.0020 (9)	0.0044 (8)	−0.0040 (9)
C8	0.0316 (10)	0.0316 (12)	0.0234 (10)	0.0038 (10)	−0.0007 (8)	−0.0027 (9)
C9	0.0310 (10)	0.0302 (12)	0.0270 (10)	0.0044 (10)	0.0029 (9)	−0.0020 (9)
C10	0.0511 (14)	0.0587 (17)	0.0299 (12)	−0.0044 (13)	−0.0007 (10)	0.0097 (12)

### Geometric parameters (Å, º)

**Table:**

S1—C9	1.738 (2)	C2—C3	1.399 (3)
S1—C10	1.791 (2)	C2—C7	1.403 (3)
S2—C1	1.645 (2)	C3—C4	1.369 (3)
N1—C8	1.360 (2)	C3—H3	0.9300
N1—C1	1.369 (3)	C4—C5	1.391 (3)
N1—H1	0.868 (10)	C4—H4	0.9300
N2—C8	1.342 (2)	C5—C6	1.369 (3)
N2—N3	1.381 (2)	C5—H5	0.9300
N2—C7	1.392 (2)	C6—C7	1.393 (3)
N3—C9	1.329 (2)	C6—H6	0.9300
N4—C8	1.319 (3)	C10—H10A	0.9600
N4—C9	1.372 (2)	C10—H10B	0.9600
C1—C2	1.471 (3)	C10—H10C	0.9600
			
C9—S1—C10	100.05 (10)	C6—C5—C4	120.89 (19)
C8—N1—C1	123.68 (17)	C6—C5—H5	119.6
C8—N1—H1	118.3 (16)	C4—C5—H5	119.6
C1—N1—H1	118.0 (16)	C5—C6—C7	119.0 (2)
C8—N2—N3	109.51 (16)	C5—C6—H6	120.5
C8—N2—C7	123.58 (17)	C7—C6—H6	120.5
N3—N2—C7	126.91 (15)	C6—C7—N2	122.05 (19)
C9—N3—N2	101.24 (15)	C6—C7—C2	121.4 (2)
C8—N4—C9	101.80 (16)	N2—C7—C2	116.50 (17)
N1—C1—C2	115.51 (18)	N4—C8—N2	111.57 (18)
N1—C1—S2	119.93 (15)	N4—C8—N1	128.80 (17)
C2—C1—S2	124.56 (17)	N2—C8—N1	119.63 (19)
C3—C2—C7	117.51 (18)	N3—C9—N4	115.88 (18)
C3—C2—C1	121.46 (19)	N3—C9—S1	124.04 (15)
C7—C2—C1	121.0 (2)	N4—C9—S1	120.09 (15)
C4—C3—C2	121.2 (2)	S1—C10—H10A	109.5
C4—C3—H3	119.4	S1—C10—H10B	109.5
C2—C3—H3	119.4	H10A—C10—H10B	109.5
C3—C4—C5	119.9 (2)	S1—C10—H10C	109.5
C3—C4—H4	120.0	H10A—C10—H10C	109.5
C5—C4—H4	120.0	H10B—C10—H10C	109.5
			
C8—N2—N3—C9	0.5 (2)	C3—C2—C7—C6	−1.4 (3)
C7—N2—N3—C9	179.49 (18)	C1—C2—C7—C6	178.41 (19)
C8—N1—C1—C2	2.8 (3)	C3—C2—C7—N2	179.17 (17)
C8—N1—C1—S2	−177.27 (16)	C1—C2—C7—N2	−1.0 (3)
N1—C1—C2—C3	178.67 (19)	C9—N4—C8—N2	1.2 (2)
S2—C1—C2—C3	−1.3 (3)	C9—N4—C8—N1	−178.6 (2)
N1—C1—C2—C7	−1.2 (3)	N3—N2—C8—N4	−1.2 (2)
S2—C1—C2—C7	178.89 (16)	C7—N2—C8—N4	179.82 (17)
C7—C2—C3—C4	0.9 (3)	N3—N2—C8—N1	178.65 (17)
C1—C2—C3—C4	−178.9 (2)	C7—N2—C8—N1	−0.4 (3)
C2—C3—C4—C5	0.1 (3)	C1—N1—C8—N4	177.7 (2)
C3—C4—C5—C6	−0.6 (3)	C1—N1—C8—N2	−2.1 (3)
C4—C5—C6—C7	0.1 (3)	N2—N3—C9—N4	0.3 (2)
C5—C6—C7—N2	−179.68 (19)	N2—N3—C9—S1	179.79 (14)
C5—C6—C7—C2	1.0 (3)	C8—N4—C9—N3	−0.9 (2)
C8—N2—C7—C6	−177.57 (19)	C8—N4—C9—S1	179.53 (15)
N3—N2—C7—C6	3.6 (3)	C10—S1—C9—N3	0.4 (2)
C8—N2—C7—C2	1.8 (3)	C10—S1—C9—N4	179.93 (17)
N3—N2—C7—C2	−177.02 (18)		

### Hydrogen-bond geometry (Å, º)

**Table:**

*D*—H···*A*	*D*—H	H···*A*	*D*···*A*	*D*—H···*A*
N1—H1···N4^i^	0.87 (1)	2.07 (1)	2.931 (2)	171 (2)

Symmetry code: (i) −*x*+1, −*y*+1, −*z*+1.
